# Epidemiology of West Nile Virus Infections in Humans, Italy, 2012–2020: A Summary of Available Evidences

**DOI:** 10.3390/tropicalmed6020061

**Published:** 2021-04-24

**Authors:** Matteo Riccò, Simona Peruzzi, Federica Balzarini

**Affiliations:** 1 Servizio di Prevenzione e Sicurezza Negli Ambienti di Lavoro (SPSAL), AUSL-IRCCS di Reggio Emilia, Via Amendola n.2, I-42122 Reggio Emilia, RE, Italy; 2 Laboratorio Analisi Chimico Cliniche e Microbiologiche, Ospedale Civile di Guastalla, AUSL-IRCCS di Reggio Emilia, I-42016 Guastalla, RE, Italy; simona.peruzzi@ausl.re.it; 3 Dipartimento P.A.A.P.S.S., Servizio Autorizzazione e Accreditamento, Agenzia di Tutela della Salute (ATS) di Bergamo, Via Galliccioli, 4, I-24121 Bergamo, BG, Italy; federica.balzarini@ats-bg.it

**Keywords:** spatiotemporal pattern, West Nile neuro-invasive disease, West Nile Virus

## Abstract

In Italy, human cases of West Nile virus (WNV) infection have been recorded since 2008, and seasonal outbreaks have occurred almost annually. In this study, we summarize available evidences on the epidemiology of WNV and West Nile neuro-invasive disease (WNND) in humans reported between 2012 and 2020. In total, 1145 WNV infection cases were diagnosed; of them 487 (42.5%) had WNND. A significant circulation of the pathogen was suggested by studies on blood donors, with annual incidence rates ranging from 1.353 (95% confidence intervals (95% CI) 0.279–3.953) to 19.069 cases per 100,000 specimens (95% CI 13.494–26.174). The annual incidence rates of WNND increased during the study period from 0.047 cases per 100,000 (95% CI 0.031–0.068) in 2012, to 0.074 cases per 100,000 (95% CI 0.054–0.099) in 2020, peaking to 0.377 cases per 100,000 (95% CI 0.330–0.429) in 2018. There were 60 deaths. Cases of WNND were clustered in Northern Italy, particularly in the Po River Valley, during the months of August (56.7%) and September (27.5%). Higher risk for WNND was reported in subjects of male sex (risk ratio (RR) 1.545, 95% CI 1.392–1.673 compared to females), and in older age groups (RR 24.46, 95% CI 15.61–38.32 for 65–74 y.o.; RR 43.7, 95% CI 28.33–67.41 for subjects older than 75 years), while main effectors were identified in average air temperatures (incidence rate ratio (IRR) 1.3219, 95% CI 1.0053–1.7383), population density (IRR 1.0004, 95% CI 1.0001–1.0008), and occurrence of cases in the nearby provinces (IRR 1.0442, 95% CI 1.0340–1.0545). In summary, an enhanced surveillance is vital for the early detection of human cases and the prompt implementation of response measures.

## 1. Introduction

West Nile virus (WNV) is a mosquito-borne RNA virus belonging to the genus *Flavivirus* (family *Flaviviridae*). In Europe, WNV is usually carried by species of the genus *Culex* (mainly *C. pipiens*, *C. peregrinus*, and *C. modestus*) and *Aedes* [1,2,3], being sustained in an enzootic “amplification” cycle between birds and mosquitoes. Even though WNV can infect large mammalians (e.g., horses), including humans, the latter represent rather incidental and dead-end hosts [2]. As a consequence, interhuman transmission usually occurs only through blood transfusion or organ transplants.

Infection in humans is usually asymptomatic, but a mild influenza-like syndrome (i.e., West Nile fever, WNF) may be observed in around 20% of all cases [4,5], while less than 1% of infected subjects develop a neuro-invasive disorder, i.e., West Nile neuro-invasive disease (WNND), which typically affects elderly, chronically ill, and immunocompromised people. Albeit infrequent, WNND is a severe condition, which can lead to the death of the patient [4,5,6,7]. Even though horse vaccines are currently available, no human vaccines or specific antiviral treatments have been to date licensed [8].

Similar to other arboviruses (e.g., tick-borne encephalitis virus or TBEV) [9,10], WNV is currently spreading in Europe [1,2,11,12,13], alongside the routes of migratory birds [1]. After the first European outbreak in Camargue, Southern France (1962), WNV has regularly involved larger areas in Eastern, Western, and Southern Europe, with a seasonal trend from April to November. Interestingly, outbreaks in humans and equines from Southern Europe usually cluster in warmer months, from July to October. Such a trend is usually explained by means of the virus introduction during bird’s spring migration, followed by its amplification in the early summer [14,15]. The simultaneous increase of vector density in warmer months will then lead to increased risk of mammalian infections.

Even though the inception of WNV occurred quite lately when compared to other European countries (i.e., 2008), currently Italy includes some among the most heavily affected areas in the European Union. Not coincidentally, in 2010, the Italian Ministry of Health implemented the first National WNV surveillance plan, which integrated veterinary, entomological, and epidemiological data in order to more efficiently monitor the spread of WNV and detect human cases of WNV infection [16,17,18,19,20]. Some earlier reports on the integrated surveillance system have been published [16,17,18,19,20], but all of them predates the transmission season of 2018, in which 1605 confirmed cases reported across European countries nearly doubled the total cases registered in the previous three years combined [21,22]. Moreover, despite the quarantine measures that various EU countries implemented during 2020, the WNV transmission season was associated with remarkable outbreaks both in previously affected and previously spared areas, with a proportion of WNND that largely exceeded the average of previous 5 years [12,23]. As a consequence, an updated and comprehensive reappraisal of surveillance data may be particularly useful to national and local health authorities for the implementation of appropriate response measures, including blood safety, and preventive campaigns focusing on vector control and communication [19,24,25].

Our aim is therefore to collect available evidence on the temporal and spatial patterns of WNV infections in Italy between 2012 and 2020, reporting appropriate insights from a country representing an example of extensive endemization of an “new” pathogen. Eventually, we will perform a comprehensive analysis on the possible influence of environmental factors on the epidemiology of WNND, focusing on climate (i.e., environmental temperatures, relative humidity, and daily precipitation) and demographics (i.e., population density, share of older age groups, and number of incident cases in the nearby areas and in the rest of EU).

## 2. Materials and Methods

Settings: Italy is administratively divided in 20 regions, in turn organized in 107 secondary administrative units composed of many municipalities (i.e., 80 ordinary provinces, 2 autonomous provinces, 4 regional decentralization entities, 6 free municipal consortia, 14 metropolitan cities, and 1 province/region: in the following text, all of them will be reported as “provinces”). With a surface of 301,340 km^2^ (116,350 sq mi), a total population of approximately 60 million inhabitants, and a population density of 201 people per square kilometer (520/sq mi), Italy is a densely but unevenly populated country in Southern Europe. Around 70% of total population may be classified as urban, a relatively low figure among high-income countries. Population density is higher in the Po River Valley (e.g., according to the 2019 census: 2098 population/km^2^ for the province of Monza and Brianza, 2004 population/km^2^ for the province of Milan, 737 population/km^2^ for the province of Varese, 436 population/km^2^ for the province of Padua, and 268 population/km^2^ for the province of Bologna), the core for both industrial and agricultural sectors, and in the metropolitan areas of Rome (i.e., 748 population/km^2^) and Naples (i.e., 2631 population/km^2^), while other vast areas are only sparsely populated (i.e., the Alps and Apennines highlands, the plateau of Basilicata, and the island of Sardinia). Italian climate is quite heterogeneous, ranging from the continental of the inland northern areas to the Mediterranean profile of coastal areas, and including humid subtropical areas in the Po River Valley. As a consequence, in Northern Italy higher population density coincides with highly irrigated agriculture areas, a condition that in turn is favorable to the ecology of mosquitoes, including vectors competent for WNV [26,27].

Data collection: In 2011, national surveillance plan for WNV infections was extensively revised, implementing the publication of periodic surveillance bulletins [17,19,28]. Periodic bulletins (available from: https://www.epicentro.iss.it/arbovirosi/bollettini; accessed on 23 April 2021) include the following data: total number of WNV infections reported during the surveillance season (i.e., 15 June to 15 November) irrespective of their actual clinical features, number of WNV infections identified in blood donors, number of WNND cases (both as a total estimate and stratified by province, age group, and month of diagnosis). According to the Italian case definition, WNND cases represent a subgroup of WNV infections including subjects with a positive PCR in urine, and/or a seroconversion test by commercially available IgM and IgG, and characterized by neurological symptoms (encephalitis, meningitis, Guillain–Barré syndrome, or acute flaccid paralysis) [17].

As data on the gender of reported cases were available only for the time period 2012–2016, and the total number of WNV incident cases were inconsistently reported by periodic bulletins, information was complemented by high-quality and highly reliable data from the ECDC Surveillance Atlas of Infectious Diseases (https://atlas.ecdc.europa.eu/public/index.aspx; accessed on 23 April 2021), and by pooling available information from regional health authorities of Lombardy, Emilia Romagna, Veneto, Friuli-Venezia-Giulia, and Piemonte (see Table A1 Appendix A). Data on new diagnoses of WNV infections on blood donors were eventually integrated through regional surveillance bulletins, and analyzed by year and region of reporting.

For each study year, data on the Italian population, in total, and for affected areas (i.e., provinces where human cases had been notified during the surveillance period) were obtained from the Italian National Statistical Institute (ISTAT; http://demo.istat.it/; accessed on 23 April 2021).

Daily meteorological data were obtained for each provincial capital from the competent Regional Environmental Protection agencies (in Italian, ARPA). Italian ARPA are the Italian environmental agencies, one for each region of Italy (excluding Trentino-Alto Adige/Süd Tirol, which has been split for the two autonomous provinces of Trento and Bolzano) (see Table A1). When more weather stations were available at the same geographical level for the same timeframe, mean values were calculated.

Statistical analysis: We performed descriptive analysis of the surveillance data, i.e., geographical and temporal distribution of human cases with WNV infection, both in general (i.e., all notified WNV infections) and then focusing on WNND, with their respective demographic characteristics (age and sex), and clinical outcome, where available. We then analyzed the corresponding annual incidence rates between 2012 and 2020. Seasonal trends were then assessed by normalizing the number of monthly reported cases (either all notified WNV infections and WNND for Italy, WNV infections for the rest of European Union) by the maximum number of new cases. Eventually, age adjusted incidence rates (ASR) for every Italian Province were calculated for WNND assuming the European standard population as reference [29].

The relationship between the number of human infections and the environmental factors was investigated through calculation of incident rate ratios (IRRs) with their correspondent 95% CI in a Poisson regression model that included as the outcome variable the yearly incident rates for WNND (assessed at provincial level). WNND cases were assumed as a proxy for all WNV infection rates in order to overcome the largely expected under-reporting [19,20]. The explanatory variables were represented by both meteorological (i.e., average daily temperature, average relative humidity, and average precipitation rates) and demographic factors (i.e., population density, number of WNND incident cases in the nearby provinces, number of WNND incident cases in the rest of EU, share of population aged 65–74 years, and share of population aged 75 years or more). In the analyses, meteorological data of the provincial capital were assigned to all WNND cases that occurred in all municipality of the province

All calculations were performed on R 4.0.3 (R Core Team (2020). R: A language and environment for statistical computing. R Foundation for Statistical Computing, Vienna, Austria. URL https://www.R-project.org/; accessed on 23 April 2021) by means of packages epiR (v. 2.0.19), EpiReport (v 1.0.1), fmsb (0.7.0), plot3d (1.3), msm (1.6.8), and sandwich (3.0–0).

Ethical approval: No ethical approval was needed for this study, as no individual data were identifiable, and only aggregated data were analyzed and presented.

## 3. Results

**Figure 1:**
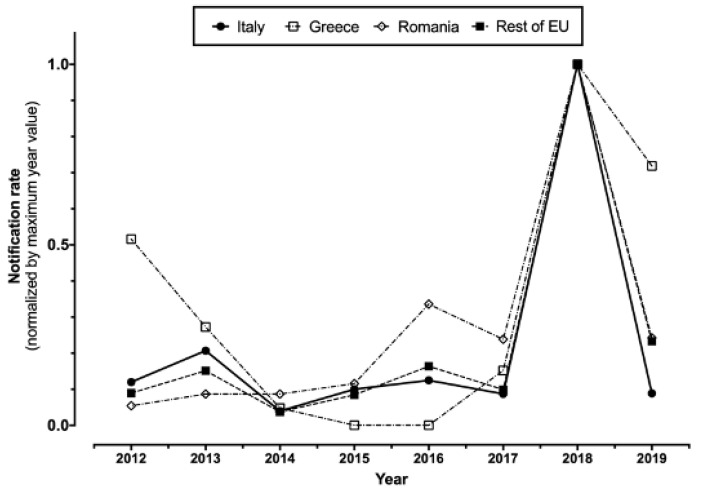
Notification rates percent normalized by maximum year value in Italy, Greece, and Romania compared to the rest of European Union (2012–2019, data were retrieved from ECDC Surveillance Atlas of Infectious Diseases (https://atlas.ecdc.europa.eu/public/index.aspx; accessed on 23 April 2021)).

In total (Table 1), 1145 WNV infections (157 of them from blood donors, i.e., 13.7%), including 487 WNND (42.5%), cases were notified in Italy between 2012 and 2020. A total of 60 WNND cases had a fatal outcome (12.3%). For two cases of WNND, no precise data on their demographics, including the month and the area of occurrence, were available at the time of the analyses. The number of reported cases peaked during 2018 for both new diagnoses of WNV infections (610, i.e., 53.3% of total sample 2012–2020), and reported cases of WNND (229, i.e., 47.0% of all retrieved cases), followed by 2013 (126 WNV infections, including 77 cases of WNND), while in the remaining timeframe the number of new incident cases remained well lower than 100 for WNV infections and 50 for WNND cases. Overall, normalized incidence rate substantially mirrored that of European Union, particularly when dealing with the peaks for 2018 and 2013, after the removal of data from Romania and Greece (i.e., two European countries are in fact characterized by high circulation of the pathogen) (Figure 1).

**Figure 2:**
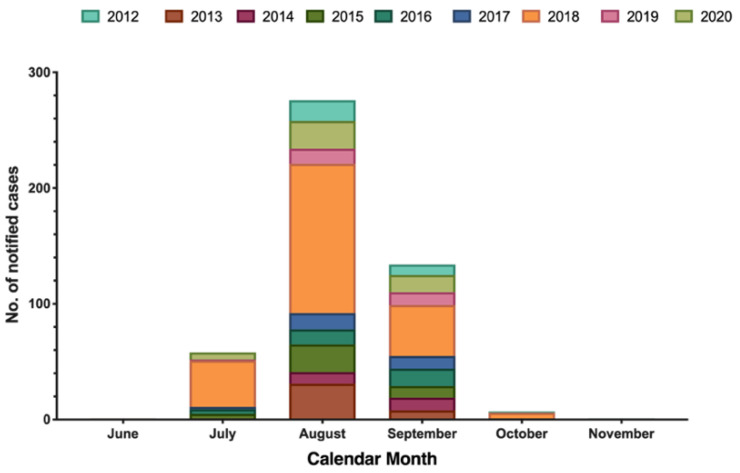
Number of laboratory diagnosed West Nile neuro-invasive disease (WNND) cases by month of symptom onset, Italy (2012–2020; no.  =  487).

The majority of new cases of WNND occurred in August (56.7%), followed by September (27.5%), July (14.0%), and October (1.4%), with only one case reported in June and November (Figure 2).

**Figure 3:**
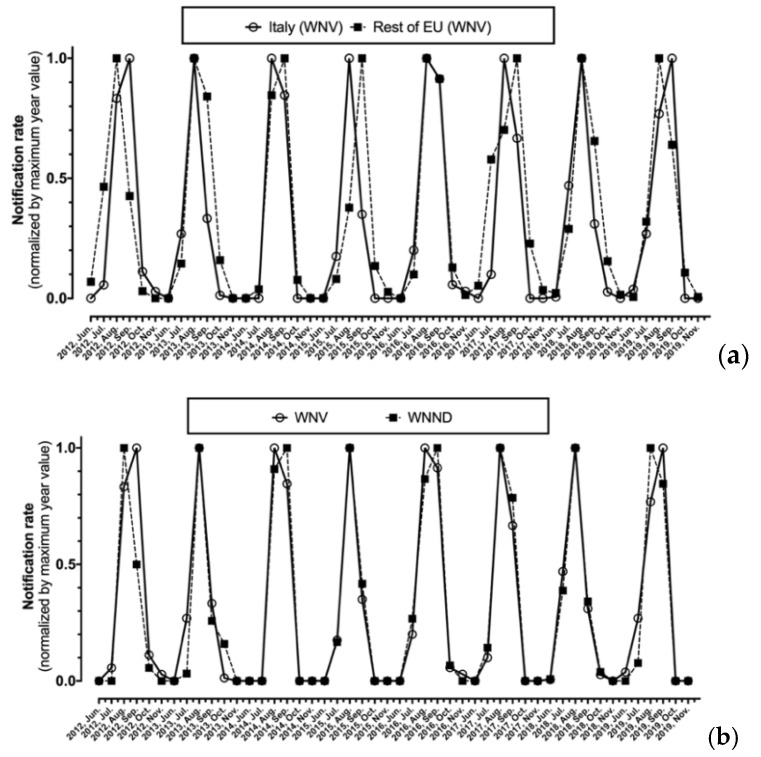
Monthly notification rate of West Nile virus (WNV) diagnoses normalized by maximum value for Italy compared to the rest of European Union (**a**), of all new diagnoses of WNV compared to corresponding new diagnoses of West Nile neuro-invasive disease (WNND) cases in Italy alone (**b**).

As shown in Figure 3, after normalization by maximum values, monthly notification rates for WNV were substantially analogous in Italy and in the rest of EU, with the annual peak ranging from August to September, being well correlated (r = 0.827, *p* < 0.001). Focusing on Italian cases, the seasonal trend for new diagnoses of WNV infection as a whole and for the subgroup of WNND were in turn well correlated (r = 0.926, *p* < 0.001) (Figure 3b).

As shown in Table 2, crude incidence rates for WNV infections ranged from 0.039 cases per 100,000 (95% CI 0.024–0.059) in 2014 to 1.009 per 100,000 (95% CI 0.930; 1.092) in 2018 (pooled estimate: 0.211 cases per 100,000, 95% CI 0.199–0.223). Similarly, also WNND incidence rates ranged from a nadir of 0.035 cases per 100,000 (95% CI 0.021–0.053) in 2014 to an apex of 0.377 per 100,000 (95% CI 0.330–0.429) in 2018, with a pooled estimate from 2012 to 2020 equals to 0.090 cases per 100,000 (95% CI 0.082–0.098). In fact, incidence rates were well correlated (r = 0.993, *p* < 0.001). Assuming the ratio WNND/WNV of 2012 as the reference year, the risk for WNND was substantially increased during the calendar year 2014 (risk ratio (RR) 2.281, 95% CI 1.643–3.166), 2015 (RR 1.624, 95% CI 1.114–2.305), and 2020 (RR 1.725, 95% CI 1.232–2.416).

**Figure 4:**
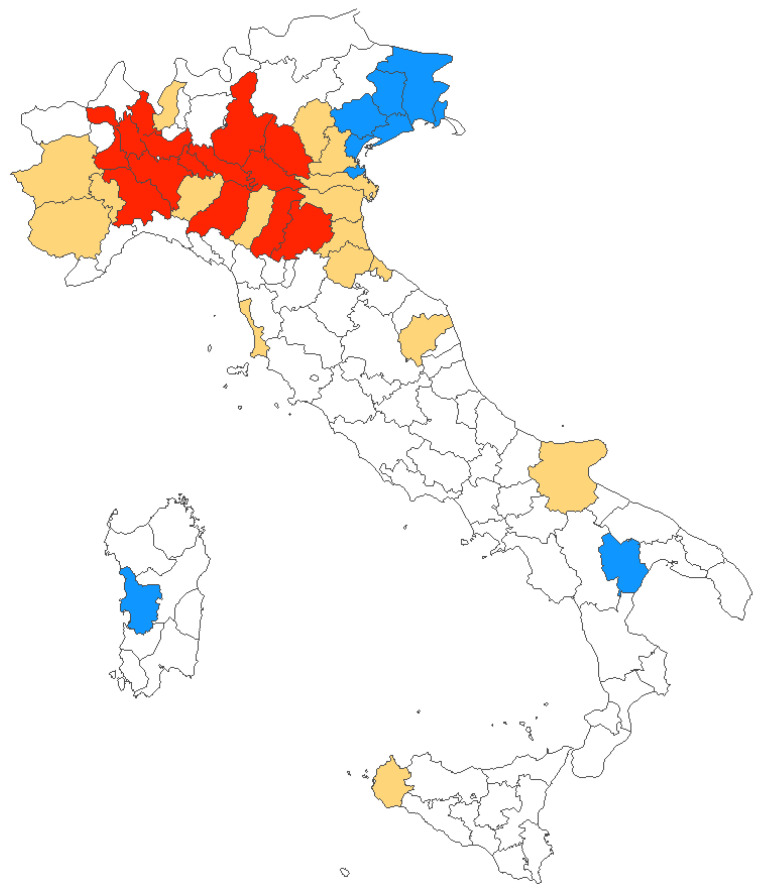
Italian provinces characterized by human WNV infections between 2012 and 2020. In blue, areas where diagnoses were originally reported in 2012; in red, all provinces with cases reported in 2020; and in yellow provinces where at least one case of WNV infections was reported between 2013 and 2019.

During the timeframe 2012–2020, mostly of reported new diagnoses of WNV infections were clustered in Northern Italy, and more precisely in provinces from the Po River Valley, with only sporadic cases from Central Italy, Southern Italy, and Sardinia. However, the number of affected provinces increased from 7 (2012) in 4 regions, to 32 (2018) in 6 regions, involving a total of 38 provinces in 11 regions. The share of the Italian population potentially exposed to the circulation of WNV similarly increased from 5.8% in 2012 to 35.8% in 2018 (19.9% in 2020) (Figure 4).

As shown in Table 3, the provinces characterized by the higher share of WNND cases during the assessed timeframe were from Emilia Romagna Region, i.e., Bologna (No. 60, 12.3%) and Modena (No. 50, 10.3%), followed by Venezia (No. 38, 7.8%), and Rovigo (No. 26, 5.3%) from the Veneto Region, and then Mantua (No. 25 5.1%) and Milan (23, 4.7%) from Lombardy. Detailed incidence rates by province during the assessed timeframe is reported in Table A2. However, as these areas are quite heterogenous in terms of resident population, the highest values of ASR were reported from Lodi (i.e., 0.926 cases per 100,000; 95% CI 0.558–1.447) and Rovigo (i.e., 0.883 cases per 100,000; 95% CI 0.532–1.379), followed by Modena (0.683 cases per 100,000; 95% CI 0.494; 0.920), Mantua (0.599 per 100,000; 95% CI 0.375–0.906), and Bologna (0.553 cases per 100,000; 95% CI 0.411–0.729), with all other provinces reporting an ASR below 0.5 cases per 100,000. In fact, a pooled ASR of 0.241 cases per 100,000 (95% CI 0.162–0.320) for affected areas was then calculated, or 0.078 cases per 100,000 (95% CI 0.007–0.149) for Italian population as a whole.

When focusing on the region of origin, the majority of cases occurred in Emilia Romagna (36.1%), followed by Veneto (25.1%), Lombardy (21.8%), Piemonte (9.7%), and Friuli-Venezia-Giulia (2.9%). Focusing on the crude incidence rates, the highest estimates were reported for Emilia Romagna (0.441, 95% CI 0.378–0.511): by assuming Emilia Romagna as the reference (Table 4), all other regions were therefore characterized by a reduced risk ratio (RR). More precisely, an RR estimate of 0.673 (95% CI 0.498–0.790), 0.289 (95% CI 0.168–0.499), 0.270 (95% CI 0.195–0.373), and 0.268 (95% CI 0.211–0.341) was calculated for Veneto, Friuli-Venezia-Giulia, Piemonte, and Lombardy, respectively.

Focusing on the demographics of reported cases, around 64.9% of new diagnoses of WNV infection occurred in males, and similarly 75.0% of all cases of WNND were of male gender, with a corresponding RR 1.545 (95% CI 1.392–1.673) for males compared to females. In this regard, it should be stressed that data availability on gender of WNND cases was limited to the time period 2012–2016 (No. = 152, i.e., 31.2% of total reported cases of WNND). The details about incident WNND cases by age group is reported, by province of occurrence, in Table A3. Briefly, around half of reported cases (i.e., 237, 48.7%) occurred in subjects aged 75 years or more, and also the incidence increased from younger age groups (i.e., <45 year-old, 0.022 per 100,000), to older ones (i.e., from 0.528 to 0.943 per 100,000, for 64–75 y.o. and 75 years or older, respectively) (Table 4).

Regarding WNV cases diagnosed from blood donors, a total of 157 out of 1145 total cases (13.7%) were retrieved for the timeframe 2012–2020 (range 0 in 2012, 30.2% in 2017), but the actual number of blood samples analyzed for WNV RNA was eventually identified only for the timeframe 2012–2018 (102 cases), and for a total reference population ranging from 4341,240 (2012) to 22,628,070 (2016) inhabitants (Table 5). Eventually, a pooled crude incidence rate of 8.337 cases per 100,000 (95% CI 6.798–10.121) was therefore calculated, peaking to 19.069 per 100,000 in 2018 (95% CI 13.494–26.174).

**Figure 5:**
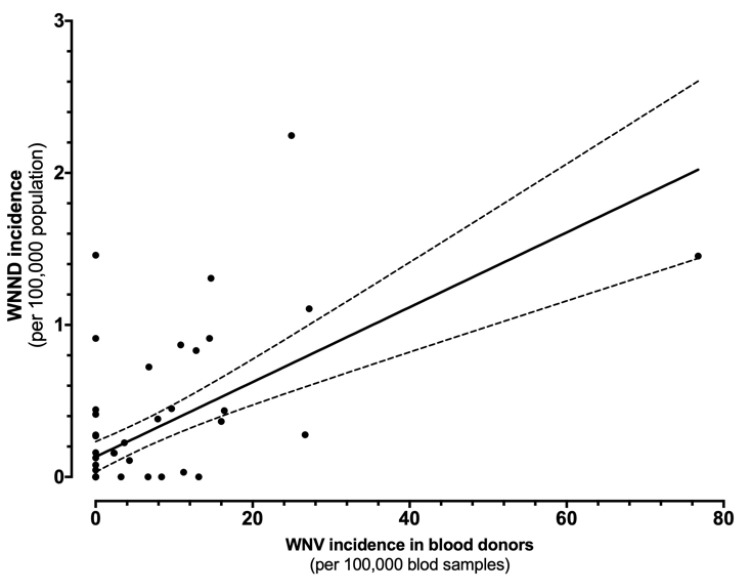
Correlation between the incidence of cases of West Nile virus (WNV) infections in blood samples and West Nile neuro-invasive diseases (WNND) in the corresponding index areas (r = 0.617, *p* < 0.001).

The direct comparison of incidence rates for WNND and WNV in blood donors is shown in Figure 5. In fact, the estimates were well correlated (r = 0.617, *p* < 0.001), i.e., greater the occurrence of WNV positive specimens among blood donors, higher the incidence of WNND cases in the same areas.

**Figure 6:**
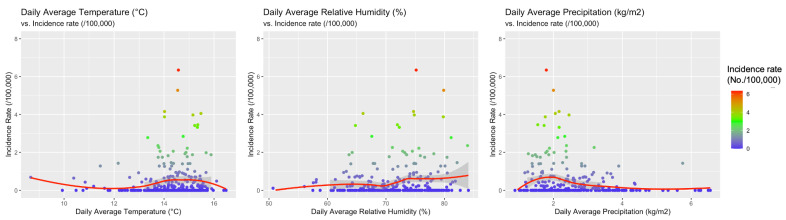
Correlation between the incidence of cases of West Nile neuro-invasive disease (WNND) in the index areas and: daily average temperatures (r = 0.0982, 95% CI −0.01754–0.2114, *p* = 0.0916), daily average precipitation (r = −0.1666, 95% CI −0.2768 to −0.0520, *p* = 0.0045), and daily average relative humidity (r = 0.1376, 95% CI 0.0224–0.2393, *p* = 0.0194).

**Figure 7:**
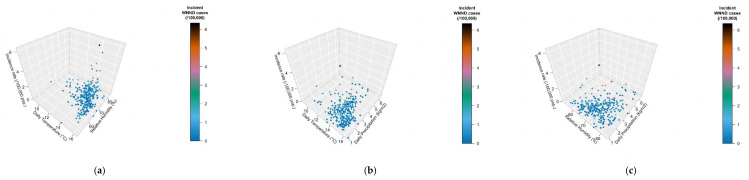
Three-dimensional representation of the incidence rate (*z*-axis), by meteorological factors, represented by daily temperature ((**a**), *x*-axis; (**b**), *x*-axis), relative humidity ((**a**), *y*-axis; (**c**), *x*-axis), and daily precipitation rate ((**b**,**c**), *y*-axis).

When meteorological factors were taken in account, incidence rates for WNND were seemly well correlated with average daily relative humidity (r = 0.1376, 95%CI 0.0224–0.2393, *p* = 0.0194), and negatively correlated with daily precipitation (r = −0.1666, 95%CI −0.2768 to −0.0520, *p* = 0.0045) while no clear correlation was identified with average daily temperatures (r = 0.0982, 95%CI −0.01754–0.2114, *p* = 0.0916). However, visual inspection of aforementioned comparisons (see Figure 6), advocated a more articulated association between incidence rates and meteorological data, with clustering of higher incidence rates for higher daily temperatures, and relative humidity, while they clustered around daily precipitation rates of 2 kg/m^2^. As shown in Figure 7, higher notification rates occurred for environmental conditions characterized by higher estimates for daily average temperatures and relative humidity, while taking in account precipitation rates resulted in a seemingly scattered distribution of the higher rates.

In fact, when climate data were included in a Poisson regression model with population density, occurrence of cases in the rest of EU and in the nearby Italian provinces, and the share of older residents over the total population (i.e., aged 65–74 years, and 75 years or more), a more distinctive pattern was identified. As shown in Table 6, an increased estimate for the average air temperature (+1.0 °C; IRR 1.3219, 95%CI 1.0053–1.7383), for the total number of incident cases in nearby provinces (+1 case/year; IRR 1.0442, 95%CI 1.0340–1.0545), and higher population density (+1 population/km^2^; IRR 1.0004, 95%CI 1.0001–1.0008) were eventually identified as effective explanatory variables.

## 4. Discussion

In recent years, outbreaks of WNV infections have hit many European countries, with the peak notification rate of 0.3 cases per 100,000 during 2018 [1,2,12,16,17,18]. Even though WNV is often described as an “emerging pathogen”, when it comes to Italy, this definition may represent a sort of misnomer: as confirmed by our analyses, large areas of Northern Italy have become endemic to this infection since early 2010s [16,17,18,30], with around one-fifth of the total population living in areas at risk. Moreover, the pooled crude incidence rate for WNV infections 2012–2020 of 0.211 cases per 100,000 (0.090 cases per 100,000 for WNND) characterizes WNV infections as a relatively rare instance, but still exceeds the EU notification rate for locally acquired cases of 0.1 cases per 100,000 [1,11,12,21,25].

**Figure A1:**
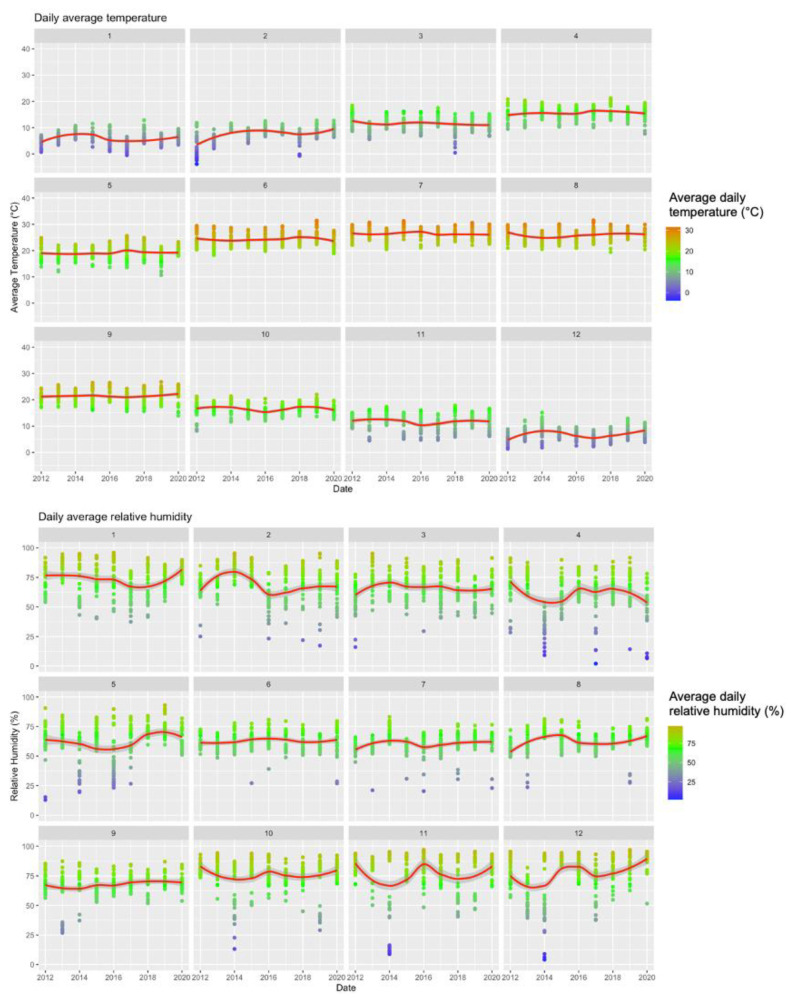

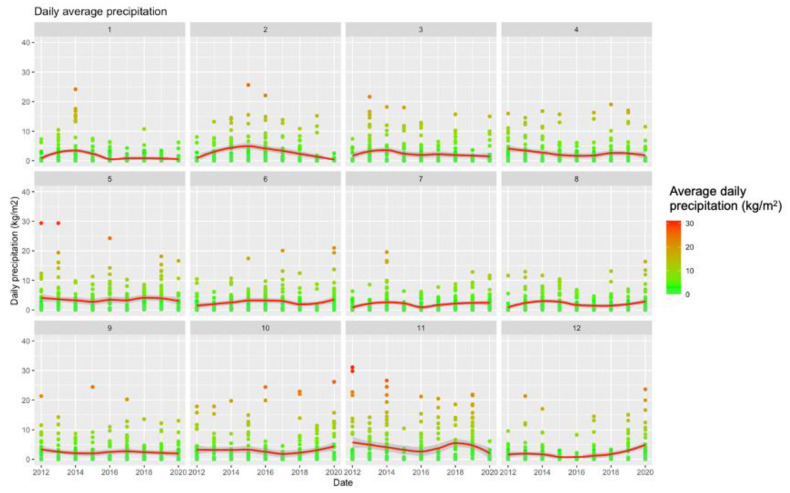
Summary of Meteorologic Data (2012–2020) Broken down by Calendar Month.

Our study also stressed substantial heterogeneities in both geographical and seasonal terms, which may contribute to our understanding of the epidemiology of WNV infections. Firstly, collected data show a strong seasonal pattern, with a sustained variability across the assessed timeframe. On the one hand, first human cases were usually observed in June, and most cases reported from July to October, peaking between August and September. On the other hand, in less than a decade, we were able to identify two incidence outliers represented by surveillance seasons 2013 and 2018, including two-thirds of all WNV infection cases, and 54.0% of all WNND cases. In particular, 2018 surveillance year was somewhat unprecedent, as it included 53.3% of all 2012–2020 WNV infection cases and 47.0% of all WNND cases, accounting for 70% of all WNV-related deaths from the inception of the active surveillance. This habit is consistent with previous reports, either from Italy and other European countries [16,25,28,31,32], and several explanations have been suggested. The more suggestive is based on the variability of climatic and meteorological conditions during the last decade, that in turn have impacted on the ecology of both vectors and reservoirs [19,20,31]. As interhuman transmission is substantially episodic, WNV depends on migratory birds for its amplification cycle, while human infections area consequence of the feeding habits of competent mosquitoes [19,20], and both factors are deeply influenced by climate and temperatures. In fact, in our estimates, incident cases for WNND found a significant effector in the environmental temperatures estimated as the average of the air temperature (i.e., IRR 1.3219, or an increase of 32.2% for a +1 °C increase in the air temperature compared to the average estimates). This result is highly consistent with some recent reports [19,20] suggesting a strong direct correlation between incidence of WNND and environmental temperatures recorded 5–6 weeks before diagnosis. On the contrary, we failed to recognize the association between precipitation and incidence rate that was similarly identified by Moirano et al., particularly for weekly total precipitation 2–3 weeks before diagnosis of WNV infections [19,20]. However, the differences in the study design must be stressed. Firstly, our analysis deliberately focused on WNND cases, a subgroup of all WNV infections that represents a reliable proxy for the burden of WNV infection, while the target population of Moirano et al. was quite heterogeneous (i.e., WNV positive blood donors, cases of WNF, and cases of WNND), being therefore only partially comparable to the present estimates. Second, while Moirano et al. preferred a case-crossover design, assessing the risk for developing WNV infection in terms of exposure lags, we retrospectively analyzed our data on the basis of the surveillance year. Even though such an approach may appear somewhat “coarser”, it represents an alternative way to cope with the considerable heterogeneity of WNND data. A considerable confirmation of the critical role played by environmental factors is represented by the notification year 2018, with an unprecedented circulation of WNV across all European countries that occurred during and after a summer season characterized by a record-breaking warmth with precipitation deficits in Central Europe [33]. Albeit summer climate conditions of the Italian peninsula were not so extreme during the summer months, warmer temperature in April-May were associated with sustained precipitations (see Figure A1), that represent an ideal setting for the amplification of competent vectors. As suggested by Marini et al. [11] after an extensive reappraisal of European, spring climate (i.e., April to May) rather than summer temperatures actually represents a critical risk factor for higher circulation of WNV during the summer season. Interestingly enough, authors also suggested that warmer temperatures during the previous winter season may be associated with a subsequent higher circulation of the pathogen in the next season, stressing a possible overwintering capacity of the vectors (either directly, or because of mutated behavior of reservoir migrating birds). In other words, climate changes may represent the primary driver of the epidemiology of WNV [11,30,34,35].

An indirect confirmation of the effect of weather and climate may be found in the similarly increased circulation of other arthropod-borne pathogens, such as Toscana virus and tick-borne encephalitis (TBE) virus during the summer season 2018 compared to the previous and following ones [36,37]. According to available figures from the Italian National Reporting System, 2018 was characterized by an estimated incidence of Toscana virus-associated meningoencephalitis of 0.149 cases per 100,000 (95% CI 0.120–0.183), compared to 0.094 per 100,000 (95% CI 0.071–0.122) in 2019, and 0.060 per 100,000 (95% CI 0.042–0.084) in 2020. During the same timeframe, notification rates for TBE in North-Eastern Italy ranged from 0.264 per 100,000 (95% CI 0.159–0.413) in 2017 to 0.523 per 100,000 (95% CI 0.385–0.742) in 2018, i.e., an estimate that nearly doubled notification rates during the time period 2000–2013 (0.38/100,000) [38].

Second, despite some foci both in humans and in animals have been reported from the regions of Marche, Apulia, Basilicata, and from Sicilia and Sardinia Islands [16,18,30,32], large areas from Central and Southern Italy remain largely “WNV-free”, or have been only episodically affected, while the large majority of all cases were identified in areas belonging to the Po River Valley, with a progressive geographical shift from east to west [19]. In fact, focusing on the reliable proxy represented by WNND, the ASR of 0.078 per 100,000 calculated at national level included foci characterized by very high circulation of the pathogen, such as the provinces of Lodi (0.926 per 100,000), Rovigo (0.883 per 100,000), and Modena (0.683 per 100,000), with incidence rates that were up to ten times greater than the national estimates. A possible explanation may be found not only in ecological characteristics of reservoirs and mosquito vectors, but also in some specificities of this geographical area. Po River Valley is quite exceptional as it represents, at the same time, the most economically developed and densely populated area of Italy, being also a very fertile, and well-irrigated territory, particularly between Eastern Lombardy and Western Emilia. Moreover, the Po River Valley is characterized by a natural waterlogging, particularly on the right side of the Po river. As a consequence, circulation of mosquito-borne pathogens has historically found a favorable ground in these Italian regions: for instance, malaria has been endemic in the Po River Valley until the 20th century, when the last marsh areas were eventually reclaimed [39], and in 2007 it hosted the first European outbreak of autochthonous cases of chikungunya [40]. Not coincidentally, Emilia Romagna Region, which is largely represented by the right side of the Po River, was characterized by higher risk for WNND during the timeframe assessed in our report. The importance of such factors is also confirmed by our analyses, as both population density, and occurrence of WNND cases in the nearby provinces were identified as significant effectors for the WNV neuro-invasive disease. Even though WNV has no inter-human spreading, higher population density increases the likelihood of being infected by pools of competent vectors, whose occurrence is in turn substantiated by higher rates in nearby areas [1,11,28,34,35].

In other words, Po River Valley should be considered at high risk for WNV and for all arthropod-borne diseases [11,41], as otherwise suggested by available data from blood donors [42]. As the majority of WNV infections occur as paucisymptomatic or even asymptomatic cases, cases of WNND are usually employed as a proxy for the broader epidemiology of WNV [19,32,43,44,45], with an eventual and extensive underestimation of actual incidence of WNV infections. Studies on blood banks represent therefore an opportunity to improve our understanding of the actual epidemiology of WNV [42], and also to avoid the potential transmission of WNV through blood transfusion [42,46]. While a previous study from the Modena Province in Emilia Romagna Region found a very low seroprevalence for WNV (i.e., 0.42%) in samples collected in 2012, subsequent and larger studies have reported a positive rate ranging from 2.9% in 2013, to 37.3% in 2018, and the latter peak quite obviously mirrored the very high circulation of the pathogen during the summer season 2018. Albeit interesting, such figures must be assessed very carefully. Even though the analysis of blood banks has become inherent to the National Surveillance System for WNV infections since its renewal in 2012 [17,18,30], precise estimates on the total number of assessed samples are not regularly available, as usually not included in the summary data. Therefore, as the availability of blood components in Italy is highly variable, with significant variation during the last years [47], without the accurate reporting of the total samples assessed for WNV, figures usually reported by National Bulletins are only limitedly informative. Furthermore, as the incidence of WNV infections is spatially variable, a different representation among sampled blood specimens of disparate areas from the very same region will eventually lead to the heterogenous rates we eventually identified.

Even though demographic data reported by the bulletins of the National Surveillance System are often discontinuous, some further insights could be drawn from the occurrence of WNV infections, and particularly of WNND cases, by gender and age groups. Actually, around two thirds of all WNV cases, and 75% of WNND cases we were able to retrieve, occurred in male subjects. This is consistent with other international reports, particularly when dealing with WNND [16,18,24,25]. While the higher rates for WNND may be explained in terms of higher susceptibility to the more severe consequence of the viral infection in male subjects, similarly to the higher occurrence among older age groups [17,18,24,25], the higher incidence of WNV infections among males, points to an increased exposure risk. Recently, the relevance of occupational exposure in the epidemiology of WNV infections has been extensively addressed, and particularly in Italy [21,22,48,49], and outdoor activities in agriculture and forestry, but also in the construction industry, are usually associated with an overrepresentation of workers of male gender. Unfortunately, as both general practitioners and occupational physicians, the medical professionals involved in the health surveillance at the workplaces [9,50], are usually characterized by little concern towards arthropod-borne pathogens, and most cases usually resolve without medical treatment, the large majority of incident cases of “summer influenza” (i.e., influenza-like illnesses occurring during the summer months, when the circulation of the influenza virus usually reaches its nadir in the Northern hemisphere) occurs without an appropriate diagnostic approach, contributing to the general underestimation of WNV epidemiology.

Albeit interesting, our data are affected by significant limits. First, we drew our estimates from national bulletins, and therefore we were able to summarize and analyze only the information that was preventively reported and analyzed by national authorities [19,20,41]. As a consequence, not only did we lack significant data about the demographics of both WNV and WNND cases, with resulting uncertainties in eventual estimates, but significant information on the reported signs and symptoms were not available to our analysis [17,18,24,30]. Second, national bulletins usually focus on WNND rather than on WNV infection cases, in order to cope with the high frequency of their underreporting [16,19]. Even though the high and even very high shares of positivity for WNV RNA among blood bank donor stress the substantial underestimation of the actual occurrence of WNV infections, the heterogeneity of corresponding raw data impairs any further generalization. Third, we are totally deprived of information about the housing of the cases and potential sources of WNV infection. This is particularly frustrating, as it is quite obvious that confirming or conversely ruling out, a significant occurrence of WNV infection in outdoor settings and in rural areas would lead to radically different public health recommendations [51,52,53]. However, as the majority of reported cases occurred in provinces characterized by highly developed agricultural settings (e.g., Lodi and Rovigo), and greater urban centers such as Milan and Turin had lower reports for both actual figures and incidence rates, it is reasonable to reaffirm a primary role for suburban and rural settings and activities. Fourth, meteorological data should be assumed as a proxy of the actual exposures: as climate factors including may strikingly fluctuate over a restricted area [54], assessment of environmental factors based on administrative unit may lack the appropriate definition we need [55,56,57]. As we were unable to retrieve georeferentiation data more accurate than the provincial level, such inaccuracies may have been significantly magnified. Moreover, it should be stressed that climate data were retrieved from regional agencies lacking a central validation, with potential dishomogeneity of retrieved data [58]. Last but not least, our data lack a significant information represented by the viral genotyping of the reported cases. According to available data, earlier reports on WNV in Italy were mostly associated with lineage 1 [45], with a subsequent cocirculation of lineage 1 and 2 between 2013 and 2018 [45,59], while the 2018 incidence peak was largely associated with infections by lineage 2 [59], which was the predominant pathogen during the European summer season 2018 [45]. In other words, without an appropriate tracking of incident cases also in terms of phylogenetic analysis, we could lose significant details, being eventually unable to properly track the actual spreading of WNV across Italy, possibly anticipating the emergence of human cases by properly identifying suitable reservoirs, and particularly certain species of migratory birds.

## 5. Conclusions

In conclusion, our study showed that northern regions of Italy, and particularly those belonging to the Po River Valley, have currently become endemic for WNV. Albeit the reported cases remain substantially rare, the significant under-reporting of total cases, stress the importance for improved surveillance by health authorities. Moreover, the epidemiology of WNND suggests that the main drivers of WNV infections may be found in demographic factors such as the population density, but also in environmental factors. In order to cope with the strikingly heterogeneities we were able to address, surveillance systems should only represent the first step of a more extensive approach, aimed to early identify and remove foci characterized by high circulation of the pathogen, with interventions focusing both on the vector and on the environment favorable to its replication.

## Figures and Tables

**Table 1:** Summary of collected data about West Nile virus (WNV) infections, with a specific focus on West Nile neuro-invasive disease (WNND) cases and related deaths in Italy, 2012–2020 (NA = not available).

Criteria	TOTAL	2020	2019	2018	2017	2016	2015	2014	2013	2012
WNV affected provinces (No./107, %)	38, 35.5%	22, 20.6%	16, 15.0%	32, 29.9%	17, 15.9%	15, 14.0%	15, 14.0%	10, 9.3%	15, 14.0%	7, 6.5%
WNV affected regions (No./20, %)	11, 55.0%	4, 20.0%	6, 30.0%	6, 30.0%	6, 30.0%	5, 25.0%	4, 20.0%	3, 15.0%	4, 20.0%	4, 20.0%
WNV infection cases (No.)	1145	68	54	610	53	76	61	24	126	73
… of male gender (No., %)	698 *, 64.9%	N.A.	35, 64.8%	374, 61.3%	39, 73.6%	53, 69.7%	55, 90.2%	18, 75.0%	77, 61.1%	47, 64.4%
… from blood donors (No., %)	157, 13.7%	16, 23.5%	7, 13.0%	68, 11.1%	16, 30.2%	22, 28.9%	13, 21.3%	3, 12.5%	12, 9.5%	0, -
WNND cases (No./All WNV cases, % (WNND/WNV))	487, 42.5%	45, 66.2%	25, 46.3%	229, 40.0%	27, 50.9%	34, 44.7%	38, 62.3%	21, 87.5%	40, 31.7%	28, 38.4%
… of male gender (No., %)	114 **, 75.0%	N.A.	N.A.	N.A.	N.A.	24, 71.0%	31, 82.0%	16, 76.0%	25, 63.0%	18, 61.0%
… occurring in (No., %)										
*June*	1, 0.2%	-	-	1, 0.4%	-	-	-	-	-	-
*July*	68, 14.0%	6, 13.3%	1, 4.0%	50, 21.8%	2, 7.4%	4, 11.8%	4, 10.5%	-	1, 2.5%	-
*August*	276, 56.7%	24, 53.3%	13, 52.0%	129, 56.3%	14, 51.9%	13, 38.2%	24, 63.2%	10, 47.6%	31, 77.2%	18, 64.3%
*September*	134, 27.5%	15, 33.3%	11, 44.0%	44, 19.2%	11, 40.7%	15, 44.1%	10, 26.3%	11, 52.4%	8, 20%	9, 32.1%
*October*	7, 1.4%	-	-	5, 2.2%	-	1, 2.9%	-	-	-	1, 3.6%
*November*	1, 0.2%	-	-	-	-	1, 2.9%	-	-	-	-
… aquired abroad (No., %)	3, 0.6%	0, -	0, -	1, 0.4%	0, -	2, 5.9%	0, -	0, -	0, -	0, -
… with fatal outcome (No., %)	60, 12.3%	5, 11.1%	5, 20.0%	42, 17.2%	N.A.	N.A.	N.A.	1, 4.8%	7, 1.7%	N.A.

* calculated for the time period 2012–2019; ** = calculated for the time period 2012–2016.

**Table 2:** Incidence of West Nile virus infections (WNV) in Italy (2012–2020), with the correspondent estimates for the subgroup of West Nile neuro-invasive disease (WNND). Crude incidence data were estimated assuming the total Italian population as the reference. Risk ratio and respective 95% confidence intervals were calculated assuming 2012 as the reference year.

Year	WNV (All Diagnoses) Incidence per 100,000 (95% Confidence Intervals)	WNND Incidence per 100,000 (95% Confidence Intervals)	Risk Ratio for WNND (95% Confidence Intervals)
2012	0.123 (0.096; 0.154)	0.047 (0.031; 0.068)	REFERENCE
2013	0.211 (0.176; 0.251)	0.067 (0.031; 0.091)	0.828 (0.562; 1.219)
2014	0.039 (0.024; 0.059)	0.035 (0.021; 0.053)	2.281 (1.643; 3.166)
2015	0.100 (0.077; 0.129)	0.064 (0.045; 0.087)	1.624 (1.114; 2.305)
2016	0.125 (0.099; 0.157)	0.056 (0.039; 0.078)	1.166 (0.795; 1.711)
2017	0.087 (0.066; 0.114)	0.045 (0.029; 0.065)	1.328 (0.897; 1.967)
2018	1.009 (0.930; 1.092)	0.377 (0.330; 0.429)	0.979 (0.719; 1.322)
2019	0.090 (0.068; 0.118)	0.042 (0.027; 0.062)	1.207 (0.802; 1.817)
2020	0.114 (0.088; 0.145)	0.074 (0.054; 0.099)	1.725 (1.232; 2.416)
POOLED	0.211 (0.199; 0.223)	0.090 (0.082; 0.098)	-

**Table 3:** Epidemiology of West Nile neuro-invasive disease (WNND) in Italy (2012–2020) by province of diagnosis. Age standardized incidence rates (ASR) and their corresponding 95% confidence intervals were calculated based on the standard European population.

Region	Province	Total Cases, %	Peak (Year)	ASR (per 100,000) (95% Confidence Interval)
Emilia Romagna	Bologna	60, 12.3%	41 (2018)	0.553 (0.411; 0.729)
	Ferrara	21, 4.3%	21 (2018)	0.476 (0.266; 0.785)
	Forlì-Cesena	2, 0.4%	2 (2018)	0.056 (0.007; 0.204)
	Modena	50, 10.3%	50 (2018)	0.683 (0.494; 0.920)
	Parma	6, 1.2%	3 (2015)	0.125 (0.041; 0.292)
	Piacenza	6, 1.2%	6 (2014)	0.194 (0.063; 0.452)
	Ravenna	14, 2.9%	13 (2018)	0.285 (0.137; 0.525)
	Reggio Emilia	16, 3.3%	6 (2013, 2018)	0.315 (0.176; 0.519)
	Rimini	1, 0.2%	1 (2015)	0.033 (0.001; 0.186)
Lombardy	Brescia	5, 1.0%	2 (2013)	0.044 (0.014; 0.103)
	Como	1, 0.2%	1 (2018)	0.019 (0.001; 0.104)
	Cremona	16, 3.3%	16 (2020)	0.433 (0.237; 0.726)
	Lodi	20, 4.1%	20 (2020)	0.926 (0.558; 1.447)
	Mantua	25, 5.1%	6 (2013, 2016)	0.599 (0.375; 0.906)
	Milan	23, 4.7%	23 (2020)	0.070 (0.043; 0.108)
	Pavia	15, 3.1%	5 (2014, 2015)	0.245 (0.127; 0.482)
	Varese	1, 0.2%	1 (2020)	0.013 (0.001; 0.070)
Piemonte	Alessandria	12, 2.5%	10 (2018)	0.235 (0.108; 0.446)
	Asti	8, 1.6%	6 (2018)	0.360 (0.145; 0.742)
	Cuneo	5, 1.0%	4 (2018)	0.076 (0.021; 0.193)
	Novara	4, 0.4%	2 (2018)	0.121 (0.033; 0.309)
	Turin	18, 3.7%	13 (2018)	0.054 (0.027; 0.097)
	Vercelli	5, 1.0%	3 (2018)	0.192 (0.040; 0.562)
Veneto	Padua	20, 4.1%	12 (2018)	0.214 (0.127; 0.339)
	Rovigo	26, 5.3%	15 (2018)	0.883 (0.532; 1.379)
	Treviso	15, 3.1%	6 (2012)	0.176 (0.096; 0.295)
	Venezia	38, 7.8%	15 (2012)	0.417 (0.285; 0.589)
	Verona	18, 3.7%	13 (2013)	0.194 (0.111; 0.314)
	Vicenza	5, 1.0%	4 (2018)	0.064 (0.021; 0.150)
Friuli Venezia Giulia	Gorizia	1, 0.2%	1 (2012)	0.080 (0.002; 0.443)
	Pordenone	7, 1.4%	4 (2018)	0.249 (0.100; 0.513)
	Udine	6, 1.2%	5 (2018)	0.104 (0.034; 0.243)
Toscana	Livorno	2, 0.4%	2 (2017)	0.066 (0.008; 0.239)
Marche	Macerata	1, 0.2%	1 (2019)	0.035 (0.001; 0.195)
Puglia	Foggia	1, 0.2%	1 (2013)	0.018 (0.001; 0.099)
Basilicata	Matera	1, 0.2%	1 (2012)	0.056 (0.001; 0.311)
Sicilia	Trapani	1, 0.2%	1 (2016)	0.026 (0.004; 0.183)
Sardegna	Oristano	9, 1.8%	4 (2017)	0.484 (0.231; 1.104)
	Not reported	2, 0.3%	-	-
POOLED	Only endemic provinces	485	228 (2018)	0.241 (0.162; 0.320)
	All Italian Population			0.078 (0.007; 0.149)

**Table 4:** Distribution of Italian West Nile neuro-invasive disease cases in Italy by region of residence and age group (2012–2020; No. = 487) and sex (2012–2016; No. = 152). A total of 485 out of 487 notified cases were included in the analyses as for 2 cases age at diagnosis was not available. For the calculation of risk ratio, age groups 0–14 and 15–44 year-old were collapsed in a single reference group.

Variable		Number of Cases (No., %)	Crude Incidence Rate (per 100,000 Population) (95% Confidence Interval)	Risk Ratio (95% Confidence Interval)
Age group	<45 years	22, 4.5%	0.022 (0.014; 0.033)	REFERENCE
	45–64	100, 20.6%	0.161 (0.131; 0.200)	7.464 (4.724; 11.8)
	64–75	126, 25.9%	0.528 (0.440; 0.629)	24.46 (15.61; 38.32)
	≥75 years	237, 48.7%	0.943 (0.827; 1.071)	43.7 (28.33; 67.41)
Gender	Female	38, 25.0%	0.169 (0.120; 0.233)	REFERENCE
	Male	114, 75.0%	0.539 (0.444; 0.646)	1.545 (1.392; 1.673)
Region	Emilia Romagna	176, 36.1%	0.441 (0.378; 0.511)	REFERENCE
	Lombardy	106, 21.8%	0.118 (0.097; 0.143)	0.268 (0.211; 0.341)
	Veneto	122, 25.1%	0.277 (0.230; 0.330)	0.673 (0.498; 0.790)
	Piemonte	47, 9.7%	0.119 (0.087; 0.159)	0.270 (0.195; 0.373)
	Friuli-Venezia-Giulia	14, 2.9%	0.127 (0.070; 0.214)	0.289 (0.168; 0.499)
	Other	22, 4.5%	0.007 (0.004; 0.010)	0.016 (0.010; 0.024)

**Table 5:** Incidence of West Nile virus (WNV) infections diagnosed in blood donors (2012–2018). Total reference population was calculated on the geographical areas where samples from donors were collected and analyzed for WNV RNA. Incidence rates were calculated on the total number of blood samples.

Year	Total Reference Population	Total Blood Samples (No.,%)	Positive Samples (No., %)	Crude Incidence Rate per 100,000 Samples (95% Confidence Interval)
2012	4,341,240	6,950, 5.7%	0, -	-
2013	6,640,626	107,118, 8.8%	12, 11.8%	11.203 (5.789; 19.569)
2014	11,524,650	221,772, 18.1%	3, 2.9%	1.353 (0.279; 3.953)
2015	19,380,719	275,759, 22.5%	16, 15.7%	5.802 (3.316; 9.942)
2016	22,628,070	310,146, 25.4%	30, 29.4%	9.673 (6.526; 13.809)
2017	8,841,367	102,408, 8.4%	3, 2.9%	2.930 (0.604; 8.561)
2018	8,828,494	199,277, 16.3%	38, 37.3%	19.069 (13.494; 26.174)
POOLED		1,223,430, 100%	102, 100%	8.337 (6.798; 10.121)

**Table 6:** Incidence rate ratios (IRR) for West Nile neuro-invasive disease (WNND) cases in Italy (2012–2018) by meteorological factors, and main characteristics of the index areas. IRR were calculated by means of a Poisson logistic regression assuming as outcome variable the annual incident cases of WNND.

Variable	Increment	IRR	95% Confidence Interval
Relative Humidity (%)	1.0%	1.0080	0.9645–1.0534
Average air Temperature (°C)	1.0 °C	1.3219	1.0053–1.7383
Daily Precipitation (kg/m^2^)	1 kg/m^2^	0.8520	0.6592–1.1012
Population density (ab/km^2^)	1 ab/km^2^	1.0004	1.0001–1.0008
Incident cases, rest of EU (cases/year)	1 case/year	1.0002	0.9995–1.0009
Incident cases, nearby provinces (cases/year)	1 case/year	1.0442	1.0340–1.0545
Residents aged 65–74 years (%)	1.0%	0.8854	0.6324–1.2340
Residents aged 75 or more (%)	1.0%	0.8986	0.7240–1.1151

## Data Availability

The data presented in this study are available on request from the corresponding author.

## Appendix A

**Table A1:** Source of Official Bulletins Included in the Study (all Link were Verified on 23 April 2021).

Document	Source	Content
Bulletins on the surveillance of Human cases of WNV, National reports	https://www.epicentro.iss.it/westnile/bollettino	Incident cases (2012–2020) WNND cases (2012–2020) Demographics (partial)
Regional bulletins on WNV from Lombardy Region	https://www.regione.lombardia.it/wps/wcm/connect/a7138e40-11b9-4273-b20e-0736be9356ee/2017_03_02+REPORT+WEST+NILE.pdf?MOD=AJPERES&CACHEID=ROOTWORKSPACE-a7138e40-11b9-4273-b20e-0736be9356ee-lGja.5v	Incident cases 2016 Data on Blood Banks for Lombardy Region (including the number of sampled specimens)
	https://www.regione.lombardia.it/wps/wcm/connect/32f70896-0d84-4b85-88b2-ed726cc5fa84/report_WND_2015.pdf?MOD=AJPERES&CACHEID=ROOTWORKSPACE-32f70896-0d84-4b85-88b2-ed726cc5fa84-lGjaM5	Incident cases 2015 Data on Blood Banks for Lombardy Region (including the number of sampled specimens)
	https://www.regione.lombardia.it/wps/wcm/connect/eceace78-7305-4b37-8c05-3d1d3b78b094/report_WND_2014.pdf?MOD=AJPERES&CACHEID=ROOTWORKSPACE-eceace78-7305-4b37-8c05-3d1d3b78b094-lGjaM9I	Incident cases 2014 Data on Blood Banks for Lombardy Region (including the number of sampled specimens)
	https://www.regione.lombardia.it/wps/wcm/connect/590291ab-5291-47ad-85cf-4a9af797826d/report_WestNileDisease_anno2013.pdf?MOD=AJPERES&CACHEID=ROOTWORKSPACE-590291ab-5291-47ad-85cf-4a9af797826d-lGjaMjb	Incident cases 2013 Data on Blood Banks for Lombardy Region (including the number of sampled specimens)
Regional bulletins on WNV from Piemonte Region	https://www.seremi.it/content/piano-nazionale-integrato-di-sorveglianza-e-risposta-ai-virus-west-nile-e-usutu-2019	Incident cases 2019 Data on Blood Banks for Lombardy Region (including the number of sampled specimens)
	https://www.seremi.it/content/piano-nazionale-integrato-di-sorveglianza-e-risposta-ai-virus-west-nile-e-usutu-2018	Incident cases 2018 Data on Blood Banks for Lombardy Region (including the number of sampled specimens)
	https://www.seremi.it/content/piano-nazionale-integrato-di-sorveglianza-e-risposta-ai-virus-west-nile-e-usutu-2017	Incident cases 2017 Data on Blood Banks for Lombardy Region (including the number of sampled specimens)
Regional Bulletins on WNV from the Emilia Romagna Region	https://salute.regione.emilia-romagna.it/normativa-e-documentazione/rapporti/malattie-infettive/EpidemiologiaWestNile2017.pdf	Summary bulletin 2008–2017 including data on incident cases (WNV positive cases/WNND), demographics, cases from blood banks
	http://archive.izsler.it/izs_bs/	Summary bulletins 2012–2014 including data on Blood Banks (with the number of sampled specimens)
Regional Bulletins on arboviruses from Veneto Region	https://sisp.aulss9.veneto.it/Arbovirosi	Incident cases 2008–2020 Data on Blood Banks for Lombardy Region (including the number of sampled specimens)
Meteorological data	https://simc.arpae.it/dext3r/	Meteorological data for Emilia Romagna Region
	https://www.arpalombardia.it/Pages/Meteorologia/Richiesta-dati-misurati.aspx	Meteorological data for Lombardy Region
	https://www.arpa.veneto.it/bollettini/storico/index.html	Meteorological data for Veneto Region
	https://www.osmer.fvg.it/archivio.php?ln=&p=dati	Meteorological data for Friuli-Venezia-Giulia Region
	http://www.arpa.piemonte.it/dati-1	Meteorological data for Piemonte Region
	http://www.sar.sardegna.it/servizi/meteo/bollsardegna_it.asp	Meteorological data for Sardegna Region
	http://www.sias.regione.sicilia.it/frameset_dati.htm	Meteorological data for Sicilia Region
	http://www.arpat.toscana.it/datiemappe#c9=banche.dati&c9=bollettini&c9=dati&c9=mappe&c0=5&b_start=0	Meteorological data for Toscana Region
	https://www.arpa.marche.it/component/k2/item/73-meteo	Meteorological data for Marche Region

**Table A2:** Crude Incidence of West Nile Neuro-Invasive Disease (WNND) Cases per Province of Notification and Year, Italy, 2012–2020 (*n* = 487 WNND Cases). Pooled Estimates were Calculated on the Total Area Characterized by at Least One WNND Case during the Observation Period; National Estimates were Calculated by Assuming the Italian Population as the Reference One.

Province	YEAR								
	2012	2013	2014	2015	2016	2017	2018	2019	2020
Bologna	-	0.101 (0.003; 0.562)	0.100 (0.003; 0.557)	0.199 (0.024; 0.719)	0.895 (0.409; 1.699)	0.396 (0.108; 1.015)	4.054 (2.909; 5.500)	-	0.196 (0.024; 0.707)
Ferrara	-	1.418 (0.460; 3.308)	-	0.282 (0.007; 1.574)	0.853 (0.176; 2.495)	-	3.458 (1.787; 6.041)	-	-
Forlì-Cesena	-	-	-	-	-	-	0.507 (0.061; 1.833)	-	-
Modena	-	0.726 (0.236; 1.695)	0.285 (0.035; 1.031)	1.139 (0.492; 2.244)	1.995 (1.091; 3.348)	0.428 (0.088; 1.251)	3.419 (2.191; 5.088)	0.566 (0.154; 1.449)	0.141 (0.004; 0.788)
Parma	-	0.232 (0.006; 1.293)	-	0.674 (0.139; 1.968)	-	-	0.222 (0.006; 1.237)	-	0.220 (0.006; 1.225)
Piacenza	-	-	0.693 (0.084; 2.504)	0.348 (0.009; 1.941)	0.348 (0.009; 1.941)	-	0.697 (0.084; 2.519)	-	-
Ravenna	-	-	-	-	-	-	3.322 (1.769; 5.681)	-	-
Reggio Emilia	-	0.766 (0.209; 1.960)	0.187 (0.005; 1.043)	0.188 (0.005; 1.045)	0.563 (0.116; 1.645)	0.563 (0.116; 1.646)	0.751 (0.205; 1.923)	-	-
Rimini	-	-	-	0.298 (0.008; 1.662)	-	-	3.322 (1.769; 5.681)	-	-
Brescia	-	0.160 (0.019; 0.579)	0.079 (0.002; 0.441)	-	-	-	0.079 (0.002; 0.441)	-	0.080 (0.002; 0.444)
Como	-	-	-	-	-	-	0.167 (0.004; 0.930)	-	-
Cremona	-	0.276 (0.007; 1.540)	0.828 (0.171; 2.421)	1.106 (0.301; 2.832)	0.277 (0.007; 1.546)	-	0.558 (0.068; 2.016)	-	1.405 (0.456; 3.278)
Lodi	-	-	0.874 (0.106; 3.154)	1.307 (0.269; 3.819)	0.436 (0.011; 2.429)	0.436 (0.011; 2.429)	0.435 (0.011; 2.425)	-	5.277 (2.727; 9.217)
Mantova	-	1.459 (0.535; 3.175)	0.482 (0.058; 1.740)	0.723 (0.149; 2.113)	1.453 (0.533; 3.163)	-	0.971 (0.265; 2.487)	0.736 (0.152; 2.140)	0.246 (0.006; 1.369)
Milano	-	0.033 (0.001; 0.181)	-	0.125 (0.034; 0.320)	0.031 (0.001; 0.174)	0.031 (0.001; 0.173)	0.185 (0.068; 0.404)	-	0.306 (0.147; 0.563)
Pavia	-	-	0.912 (0.296; 2.128)	0.911 (0.296; 2.126)	-	-	0.183 (0.005; 1.021)	-	0.740 (0.202; 1.895)
Varese	-	-	-	-	-	-	-	-	0.113 (0.003; 0.630)
Alessandria	-	-	-	-	-	-	2.358 (0.131; 4.336)	-	0.479 (0.058; 1.731)
Asti	-	-	-	-	-	0.923 (0.112; 3.334)	2.779 (1.020; 6.049)	-	-
Cuneo	-	-	-	-	-	-	0.680 (0.185; 1.740)	0.170 (0.004; 0.949)	-
Novara	-	-	-	-	0.270 (0.007; 1.504)	-	0.541 (0.066; 1.955)	-	0.274 (0.007; 1.527)
Torino	-	-	-	0.044 (0.001; 0.243)	-	-	0.573 (0.305; 0.980)	0.179 (0.049; 0.457)	-
Vercelli	-	-	-	-	-	-	1.741 (0.359; 5.088)	0.587 (0.015; 3.268)	0.590 (0.015; 3.289)
Padova	-	0.108 (0.003; 0.600)	0.107 (0.003; 0.595)	-	-	-	1.281 (0.662; 0.238)	0.642 (0.236; 1.398)	-
Rovigo	-	2.061 (0.669; 4.811)	-	0.412 (0.010; 2.297)	0.831 (0.101; 3.004)	1.257 (0.259; 3.675)	6.345 (3.551; 10.465)	-	-
Treviso	0.685 (0.251; 1.491)	0.454 (0.124; 1.162)	-	-	-	0.226 (0.027; 0.815)	0.338 (0.070; 0.988)	-	-
Venezia	1.772 (0.992; 2.923)	0.236 (0.029; 0.852)	-	-	-	0.234 (0.028; 0.846)	1.874 (1.071; 3.044)	0.235 (0.028; 0.849)	0.118 (0.003; 0.656)
Verona	-	0.110 (0.003; 0.614)	0.108 (0.003; 0.604)	-	0.108 (0.003; 0.604)	-	1.409 (0.750; 2.409)	0.108 (0.003; 0.604)	0.108 (0.003; 0.603)
Vicenza	-	-	-	-	-	-	0.463 (0.126; 1.186)	0.117 (0.003; 0.650)	-
Gorizia	0.715 (0.018; 3.982)	-	-	-	0.578 (0.015; 3.220)	-	-	-	-
Pordenone	0.644 (0.078; 2.326)	-	-	-	-	-	1.282 (0.349; 3.282)	0.322 (0.008; 1.794)	-
Udine	0.187 (0.005; 1.042)	-	-	-	-	-	0.944 (0.307; 2.204)	-	-
Livorno	-	-	-	-	-	0.593 (0.072; 2.142)	-	-	-
Macerata	-	-	-	-	-	-	-	0.319 (0.008; 1.780)	-
Foggia	-	0.159 (0.004; 0.887)	-	-	-	-	-	-	-
Matera	0.500 (0.013; 2.785)	-	-	-	-	-	-	-	-
Trapani	-	-	-	-	0.229 (0.006; 1.279)	-			
Oristano	1.222 (0.148; 4.414)	-	-	-	-	2.488 (0.678; 6.371)	1.884 (0.389; 5.506)	-	-
National estimate	0.047 (0.031; 0.068)	0.067 (0.031; 0.091)	0.035 (0.021; 0.053)	0.064 (0.045; 0.087)	0.056 (0.039; 0.078)	0.045 (0.029; 0.065)	0.377 (0.330; 0.429)	0.042 (0.027; 0.062)	0.074 (0.054; 0.099)

**Table A3:** Epidemiology of West Nile Neuro-Invasive Disease (WNND) in Italy (2012–2020) by Age Groups According to Official Surveillance Data of Italian National Health Institute (in Italian, ISS). Note: ASR = Age Standardized Incidence Rate.

Province	Total Cases, %	Peak (Year)	Incidence (per 100,000 Person-Years)						ASR (per 100,000)
			Pooled	Age Group (Year)					
				0–14	15–44	45–64	65–74	≥75	
Emilia Romagna									
Bologna	60	41 (2018)	0.664 (0.507; 0.855)	-	0.106 (0.022; 0.309)	0.344 (0.157; 0.653)	1.872 (1.127; 2.923)	2.459 (1.647; 3.531)	0.553 (0.411; 0.729)
Ferrara	21	21 (2018)	0.666 (0.413; 1.019)	-	0.103 (0.003; 0.572)	0.103 (0.003; 0.574)	0.985 (0.269; 2.523)	3.320 (1.858; 5.476)	0.476 (0.266; 0.785)
Forlì-Cesena	2	2 (2018)	0.056 (0.007; 0.204)	-	0.083 (0.002; 0.460)	0.098 (0.003; 0.545)	-	-	0.056 (0.007; 0.204)
Modena	50	50 (2018)	0.794 (0.589; 1.047)	-	0.045 (0.001; 0.025)	0.720 (0.383; 1.231)	1.310 (0.599; 2.487)	3.714 (2.448; 5.404)	0.683 (0.494; 0.920)
Parma	6	3 (2015)	0.150 (0.055; 0.326)	-	-	-	0.463 (0.056; 1.671)	0.809 (0.220; 2.071)	0.125 (0.041; 0.292)
Piacenza	6	6 (2014)	0.233 (0.085; 0.506)	-	0.116 (0.003; 0.645)	-	0.341 (0.009; 1.899)	1.165 (0.317; 2.982)	0.194 (0.063; 0.452)
Ravenna	14	13 (2018)	0.399 (0.218; 0.670)	-	-	0.097 (0.002; 0.415)	0.759 (0.156; 2.217)	2.096 (1.005; 3.855)	0.285 (0.137; 0.525)
Reggio Emilia	16	16 (2013, 2018)	0.336 (0.192; 0.545)	-	-	0.075 (0.002; 0.415)	1.459 (0.587; 3.006)	1.538 (0.664; 3.031)	0.315 (0.176; 0.519)
Rimini	1	1 (2015)	0.033 (0.001; 0.186)	-	-	-	0.305 (0.008; 1.699)	-	0.033 (0.001; 0.186)
Lombardia									
Brescia	5	2 (2013)	0.044 (0.014; 0.103)	-	-	0.031 (0.001; 0.173)	0.170 (0.021; 0.613)	0.174 (0.021; 0.629)	0.044 (0.014; 0.103)
Como	1	1 (2018)	0.019 (0.001; 0.104)	-	-	-	0.170 (0.004; 0.948)	-	0.019 (0.001; 0.104)
Cremona	16	16 (2020)	0.495 (0.283; 0.804)	-	-	0.635 (0.233; 1.382)	1.091 (0.297; 2.793)	1.547 (0.568; 3.367)	0.433 (0.237; 0.726)
Lodi	20	20 (2020)	0.975 (0.596; 1.506)	-	0.135 (0.003; 0.754)	0.502 (0.104; 1.466)	2.313 (0.751; 5.397)	5.298 (2.645; 9.480)	0.926 (0.558; 1.447)
Mantova	25	6 (2013, 2016)	0.680 (0.440; 1.004)	-	-	0.470 (0.153; 1.098)	2.460 (1.180; 4.524)	2.396 (1.149; 4.407)	0.599 (0.375; 0.906)
Milano	23	23 (2020)	0.080 (0.051; 0.120)	-	-	0.073 (0.027; 0.159)	0.254 (0.110; 0.501)	0.274 (0.126; 0.521)	0.070 (0.043; 0.108)
Pavia	15	15 (2014, 2015)	0.306 (0.172; 0.505)	0.163 (0.004; 0.910)	-	0.206 (0.043; 0.603)	0.182 (0.005; 1.012)	1.622 (0.778; 2.983)	0.245 (0.127; 0.482)
Varese	1	1 (2020)	0.013 (0.001; 0.070)	-	-	-	0.112 (0.003; 0.622)	-	0.013 (0.001; 0.070)
Piemonte									
Alessandria	12	10 (2018)	0.314 (0.162; 0.548)	-	0.085 (0.002; 0.471)	0.257 (0.053; 0.751)	0.619 (0.128; 1.810)	0.890 (0.289; 2.078)	0.235 (0.108; 0.446)
Asti	8	6 (2018)	0.411 (0.178; 0.811)	-	0.160 (0.004; 0.894)	0.173 (0.004; 0.963)	1.280 (0.264; 3.741)	1.129 (0.233; 3.300)	0.360 (0.145; 0.742)
Cuneo	5	4 (2018)	0.094 (0.031; 0.220)	-	-	0.065 (0.002; 0.364)	0.502 (0.103; 1.466)	0.153 (0.004; 0.850)	0.076 (0.021; 0.193)
Novara	4	2 (2018)	0.121 (0.033; 0.309)	-	-	0.202 (0.024; 0.728)	0.544 (0.066; 1.964)	-	0.121 (0.033; 0.309)
Torino	18	13 (2018)	0.088 (0.052; 0.140)	-	-	0.050 (0.010; 0.146)	0.123 (0.025; 0.361)	0.463 (0.239; 0.808)	0.054 (0.027; 0.097)
Vercelli	5	3 (2018)	0.321 (0.104; 0.745)	-	-	0.634 (0.131; 1.852)	0.525 (0.013; 2.924)	0.447 (0.011; 2.490)	0.192 (0.040; 0.562)
Veneto									
Padova	20	12 (2018)	0.238 (0.145; 0.368)	-	0.102 (0.021; 0.299)	0.160 (0.044; 0.409)	0.445 (0.121; 1.140)	0.988 (0.452; 1.875)	0.214 (0.127; 0.339)
Rovigo	26	15 (2018)	1.208 (0.789; 1.770)	-	-	0.742 (0.241; 1.732)	2.993 (1.368; 5.681)	4.348 (2.247; 7.595)	0.883 (0.532; 1.379)
Treviso	15	6 (2012)	0.189 (0.106; 0.311)	-	0.071 (0.009; 0.260)	0.215 (0.070; 0.501)	0.356 (0.073; 1.040)	0.600 (0.195; 1.400)	0.176 (0.096; 0.295)
Venezia	38	15 (2012)	0.495 (0.350; 0.680)	0.102 (0.003; 0.568)	0.079 (0.010; 0.284)	0.300 (0.121; 0.619)	0.773 (0.311; 1.592)	2.304 (1.426; 3.522)	0.417 (0.285; 0.589)
Verona	18	18 (2013)	0.218 (0.129; 0.344)	-	-	0.253 (0.093; 0.552)	0.231 (0.028; 0.834)	1.119 (0.537; 2.057)	0.194 (0.111; 0.314)
Vicenza	5	4 (2018)	0.064 (0.021; 0.150)	-	0.036 (0.001; 0.202)	0.044 (0.001; 0.246)	0.122 (0.003; 0.682)	0.249 (0.030; 0.901)	0.064 (0.021; 0.150)
Friuli Venezia Giulia									
Gorizia	1	1 (2012)	0.080 (0.002; 0.443)	-	-	-	-	0.578 (0.015; 3.220)	0.080 (0.002; 0.443)
Pordenone	7	4 (2018)	0.249 (0.100; 0.513)	-	0.103 (0.003; 0.572)	0.494 (0.134; 1.264)	0.625 (0.076; 2.258)	-	0.249 (0.100; 0.513)
Udine	6	5 (2018)	0.125 (0.046; 0.272)	-	-	0.139 (0.017; 0.500)	0.165 (0.003; 0.918)	0.497 (0.103; 1.453)	0.104 (0.034; 0.243)
Toscana									
Livorno	2	2 (2017)	0.066 (0.009; 0.239)	-	-	-	0.515 (0.062; 1.861)	-	0.066 (0.008; 0.239)
Marche									
Macerata	1	1 (2019)	0.035 (0.001; 0.195)	-	-	-	-	-	0.035 (0.001; 0.195)
Puglia									
Foggia	1	1 (2013)	0.018 (0.001; 0.099)	-	-	-	0.175 (0.004; 0.974)	-	0.018 (0.001; 0.099)
Basilicata									
Matera	1	1 (2012)	0.056 (0.001; 0.311)	-	-	0.197 (0.005; 0.197)	-	-	0.056 (0.001; 0.311)
Sicilia									
Trapani	1	1 (2016)	0.026 (0.001; 0.144)	-	0.070 (0.002; 0.387)		-	-	0.026 (0.004; 0.183)
Sardegna									
Oristano	9	4 (2017)	0.622 (0.284; 1.180)	-	-		0.564 (0.014; 3.142)	3.290 (1.207; 7.161)	0.484 (0.231; 1.104)
POOLED	485	229 (2018)	0.228 (0.209; 0.250)	0.070 (0.001; 0.025)	0.027 (0.017; 0.042)	0.161 (0.13; 0.196)	0.528 (0.440; 0.629)	0.943 (0.827; 1.071)	0.241 (0.162; 0.320)
